# Search strategy analysis of Tg4-42 Alzheimer Mice in the Morris Water Maze reveals early spatial navigation deficits

**DOI:** 10.1038/s41598-022-09270-1

**Published:** 2022-03-31

**Authors:** Nadine Curdt, Franziska W. Schmitt, Caroline Bouter, Trendelina Iseni, Hanna C. Weile, Berfin Altunok, Nicola Beindorff, Thomas A. Bayer, Matthew B. Cooke, Yvonne Bouter

**Affiliations:** 1 Department of Psychiatry and Psychotherapy, Division of Molecular Psychiatry, Georg-August-University Göttingen, University Medicine Göttingen, 37075 Göttingen, Germany; 2 Department of Nuclear Medicine, University Medical Center Göttingen (UMG), Georg-August-University, Göttingen, Germany; 3 Berlin Experimental Radionuclide Imaging Center (BERIC), Charité—University Medicine Berlin, Berlin, Germany; 4 Djavad Mowafaghian Centre for Brain Health, University of British Columbia, Vancouver, BC V6T 1Z3 Canada; 5 Department of Psychology, University of British Columbia, Vancouver, BC V6T 1Z4 Canada

**Keywords:** Alzheimer's disease, Neuroscience, Spatial memory

## Abstract

Spatial disorientation is one of the earliest symptoms in Alzheimer’s disease and allocentric deficits can already be detected in the asymptomatic preclinical stages of the disease. The Morris Water Maze (MWM) is used to study spatial learning in rodent models. Here we investigated the spatial memory of female 3, 7 and 12 month-old Alzheimer Tg4-42 mice in comparison to wild-type control animals. Conventional behavior analysis of escape latencies and quadrant preference revealed spatial memory and reference memory deficits in female 7 and 12 month-old Tg4-42 mice. In contrast, conventional analysis of the MWM indicated an intact spatial memory in 3 month-old Tg4-42 mice. However, a detailed analysis of the swimming strategies demonstrated allocentric-specific memory deficits in 3 month-old Tg4-42 mice before the onset of severe memory deficits. Furthermore, we could show that the spatial reference memory deficits in aged Tg4-42 animals are caused by the lack of allocentric and spatial strategies. Analyzing search strategies in the MWM allows to differentiate between hippocampus-dependent allocentric and hippocampus-independent egocentric search strategies. The spatial navigation impairments in young Tg4-42 mice are well in line with the hypometabolism and synaptic deficits in the hippocampus. Therefore, analyzing search strategies in the Tg4-42 model can be a powerful tool for preclinical drug testing and identifying early therapeutic successes.

## Introduction

Alzheimer’s disease (AD), the most common form of dementia, is characterized by progressive cognitive decline and neurodegeneration. Spatial disorientation, often referred to as wandering, is one of the earliest symptoms of memory deficits in AD^1–4^. Patients with mild cognitive impairment (MCI) and patients in the early stages of symptomatic AD often get lost in unfamiliar as well as familiar places^2,5^. Furthermore, several studies show that deficits in spatial navigation and orientation are more specific in distinguishing AD from other forms of dementia than episodic memory deficits^6,7^. Some studies even suggest that disorientated patients are more likely to progress from MCI to AD^8,9^. Impairments in spatial navigation may therefore serve as an early indicator of AD.

The Morris Water Maze (MWM), which was originally introduced by Richard G. Morris in 1983, is the gold standard memory test in rodents and a popular tool in the cognitive assessment of AD mice^10^. The test is routinely used in studies investigating the disease pathogenesis and progression, as well as in studies testing efficacy of therapeutic interventions^11,12^. Learning in the MWM is quantified using specific parameters such as latency to reach the platform, average distance from the platform, path efficacy, or swimming distance. In addition, swimming speed or immobility are often used as control variables. However, such single measures cannot reflect the complexity of swimming and search behavior and fail to provide a detailed picture of how an animal solves a spatial navigation task^13,14^. Therefore, the aim of the current study was to identify possible spatial navigation deficits in Tg4-42 mice by analyzing the search strategies in the MWM.

**Figure 1:**
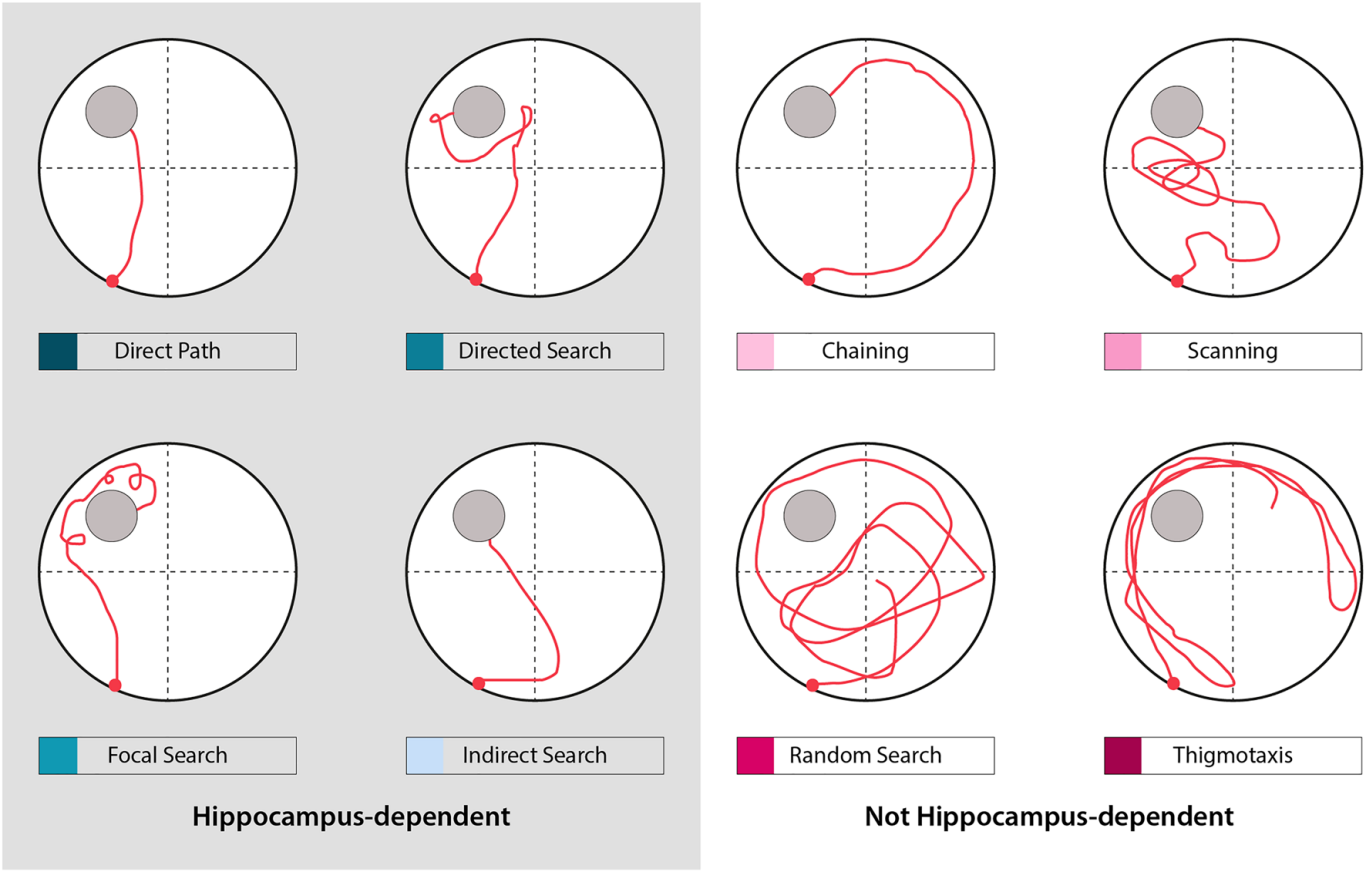
Representative examples of possible search strategies. Search strategies used by mice to locate the hidden platform can be divided into hippocampus-dependent and not hippocampus-dependent strategies. Pathfinder categorizes each trial according to 1 of 8 possible strategies. Spatial strategies include ‘direct path’, ‘directed search’, ‘focal search’ and ‘indirect search’. ‘Chaining’, ‘scanning’, ‘random search’ and ‘thigmotaxis’ are considered as non-spatial strategies.

The Tg4-42 line is a unique mouse model because, unlike most AD models, it does not overexpress mutant forms of human amyloid precursor protein (APP) or mutations linked to autosomal-dominant forms of AD and therefore may be more relevant to the sporadic form of AD. Tg4-42 mice overexpress Aβ4-42 under the control of the murine neuron-specific Thy1-promoter, leading to intracellular Aβ accumulation in the brain^15^. Therefore, the Tg4-42 mouse represents a unique model system for studying the effect of chronic exposure of Aβ4-42 in the mouse brain. Aβ4-42 peptides are highly abundant in the brain of AD patients and were among the first Aβ peptides that have been identified in AD patients^16–18^. In Tg4-42 mice intracellular Aβ4-42 accumulation is accompanied by micro- and astrogliosis that is most pronounced in the hippocampus^15,19^. Furthermore, the model is characterized by age-dependent severe synaptic impairments and neuron loss albeit without plaque formation^19–22^. In addition, age-dependent memory and behavioral deficits starting at 6 months of age were described in Tg4-42 mice. Impairments in object recognition memory and spatial memory as well as motor deficits and reduced anxiety behavior have been reported in the Tg4-42 line^20,21,23,24^. Several studies described that Tg4-42 exhibit deficits in spatial memory in the MWM by analyzing escape latencies and goal preference. However, these measures cannot identify subtle behavioral deficits with high sensitivity as they focus solely on endpoints. Conventional behavior analysis using the Tg4-42 model in the MWM indicated that spatial memory is intact before the age of 6 months^15,20,21^. However, the specific mechanism and strategies used by Tg4-42 mice in the MWM to locate the goal remain unclear. Therefore, the aim of the present work was to perform a detailed analysis of swimming strategies to better understand the known behavioral impairments in Tg4-42 and to identify possible early deficits in spatial navigation (Fig. 1). In the current study, conventional escape latencies as well as swimming strategies of 3, 7 and 12 month-old homozygous Tg4-42 mice in the MWM were analyzed using the Pathfinder program^14^.

## Results

Spatial reference memory was tested in homozygous 3, 7 and 12 month-old Tg4-42 and aged-matched WT mice in the MWM.

### Cued training reveals intact vision and motor ability of Tg4-42 mice to perform the test

Testing began with 3 days of cued training to rule out possible motor or sensory deficits that could affect the performance of mice. WT and Tg4-42 female mice showed progressively decreased escape latencies over time independent of their age (Fig. S1A,C,E: two-way repeated measures ANOVA, *days*: **3m** F(2,76) = 45.15, *p* < 0.001; **7m** F(2,70) = 26.97, *p* < 0.001; **12m** F(2,63) = 26.66, *p* < 0.001). While 12 month old Tg4-42 demonstrated decreased escape latencies over the 3 days of training, they showed an overall worse performance than WT animals (Fig. S1A,C,E: two-way repeated measures ANOVA, genotype: **3m** F(1,24) = 1.111, *p* = 0.3023; **7m** F(1,22) = 3.568, *p* = 0.0722; **12m** F(1,19) = 46.61, *p* < 0.0001). 12 month-old Tg4-42 showed longer escape latencies on the first and second day of cued training (Fig. S1E: two-way repeated measures ANOVA, day1: *p* = 0.0023, day2: *p* = 0.0021).

In addition, 7 month-old transgenic mice took significantly longer to reach the cued platform on the last day of cued training than WT mice (Fig. S1C: two-way repeated measures ANOVA, day3: *p* = 0.0149). Swimming speed did not differ between WT and Tg4-42 mice at any of the ages tested (Fig. S1B,D,F: two-way repeated measures ANOVA, genotype: **3m** F(1,24) = 4.171, *p* = 0.0523; **7m** F(1,22) = 2.998, *p* = 0.0974; **12m** F(1,19) = 3.107, *p* = 0.941). Overall, the cued training demonstrated that all mice had the abilities to perform the test.

### Age-dependent spatial learning deficits of Tg4-42 mice in the acquisition training

**Figure 2:**
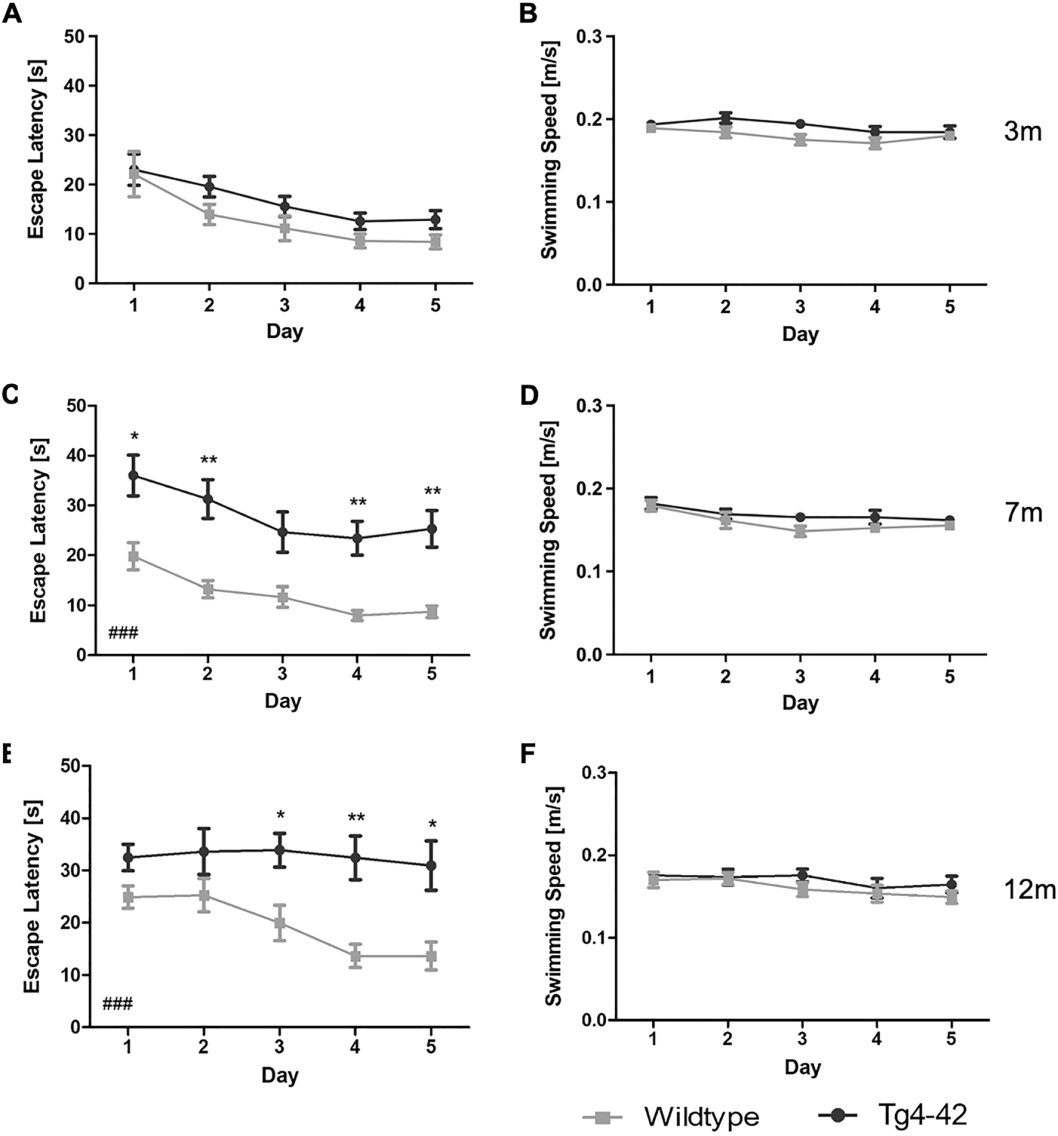
Tg4-42 mice display age-related spatial learning deficits in the acquisition training. Homozygous female Tg4-42- and WT mice were tested at 3m (**A**, **B**), 7m (**C**, **D**) and 12m (**E**–**F**). At 3m, escape latencies did not differ significantly between Tg4-42 and WT mice (**A**). In contrast, 7m (**C**) and 12m (**E**) Tg4-42 mice required significantly more time to reach the goal platform than same-aged WT mice. Swimming speed (**B**, **D**, **F**) did not differ between Tg4-42 and WT animals independent of age. Two-way repeated measures ANOVA followed by Bonferroni multiple comparisons; n = 9–14. All Data presented as mean ± S.E.M. ANOVA: ^###^*p* < 0.001; Bonferroni: *p < 0.05, **p < 0.01, ***p < 0.001.

During the subsequent acquisition training, the learning ability to locate a hidden platform using distal and proximal cues was tested in all mice. The escape latency did not differ between 3 month-old Tg4-42 mice and same-aged WT animals across the 5 days of acquisition training (Fig. 2A: two-way repeated measures ANOVA, *genotype*: **3m** F(1,24) = 4.125, *p* = 0.0535). In contrast, a significant main effect of genotype was found in 7 and 12 month-old mice, with WT mice showing lower escape latencies than Tg4-42 animals of the same age (Fig. 2C,E: two-way repeated measures ANOVA, *genotype*: **7m** F(1,22) = 24.52, *p* < 0.001; **12m** F(1,19) = 19.0, *p* < 0.001). 7 month-old WT mice showed a better performance than Tg4-42 animals over the 5 days of testing (Fig. 2C: Bonferroni multiple comparisons, *day 1:* *p* < 0.05; *day 3:* *p* = 0.0588; *day 2, day 4, day 5:* *p* < 0.01). In addition, 12 month-old Tg4-42 mice required significantly more time compared to WT animals to find the hidden platform on the last 3 days of the acquisition training (Fig. 2E: Bonferroni multiple comparisons, *day 3, day 5*: *p* < 0.05; *day 4*: *p* < 0.01). Swimming speed did not differ between WT and Tg4-42 mice at any of the ages tested (Fig. 2B,D,F, two-way repeated measures ANOVA, *genotype:* **3m** F(1,24) = 3.204, *p* = 0.861; **7m** F(1,22) = 2.389, *p* = 0.1365; **12m** F(1,19) = 0.7599, *p* = 0.3942).

In summary, Tg4-42 mice showed age-dependent spatial learning deficits in the acquisition training.

### Age-dependent spatial reference memory deficits in Tg4-42 mice in the probe trial

Twenty-four hours after the last day of acquisition training, a probe trial was performed to assess spatial reference memory.

**Figure 3:**
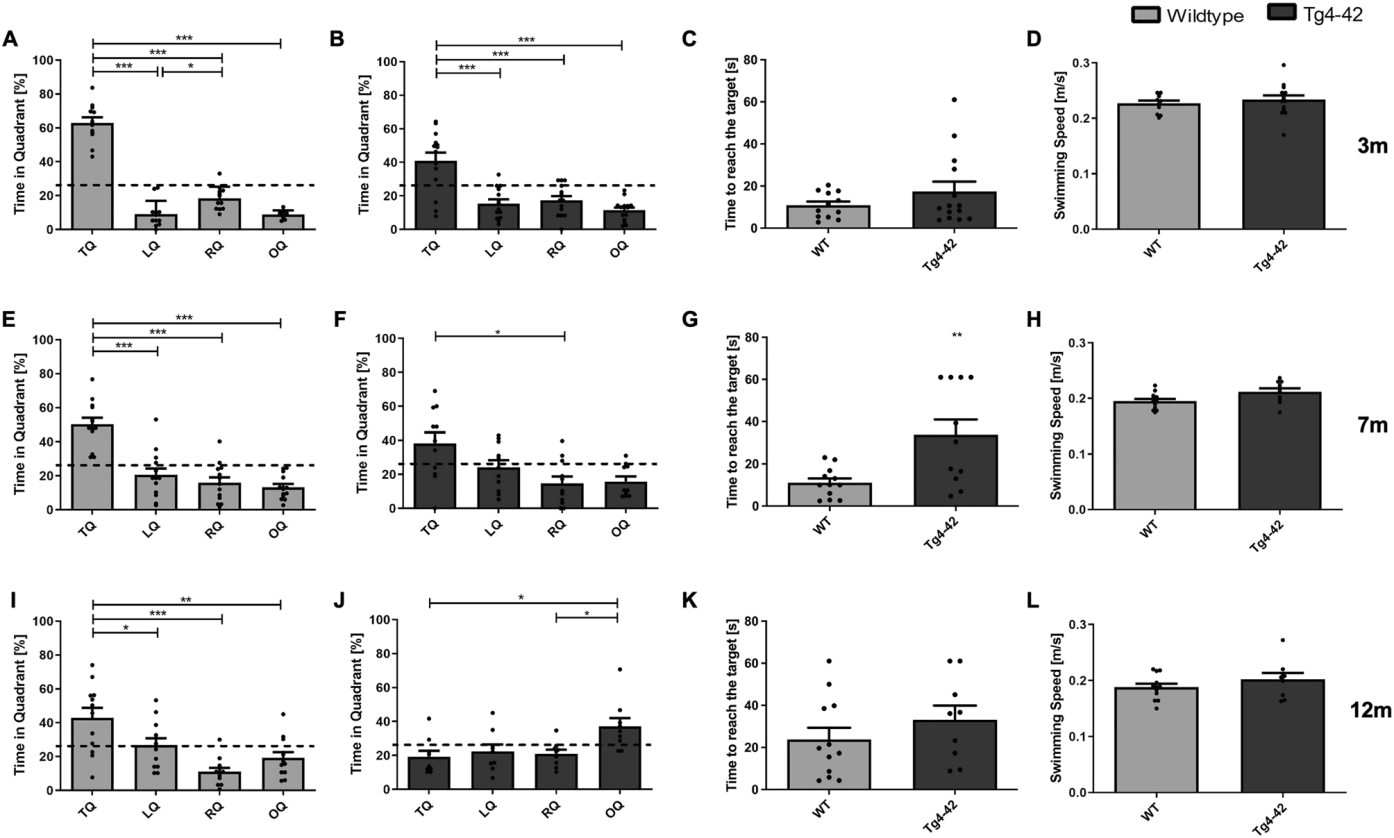
Tg4-42 mice show age-dependent deficits in spatial reference memory in the probe trial. Homozygous female Tg4-42 and WT mice were tested at 3m (**A**–**D**), 7m (**E**–**H**) and 12m (**I**–**L**). 3m WT (**A**) and Tg4-42 mice (**B**) showed a significant preference for the target quadrant. Time to reach the target did not differ between genotypes at this age (**C**). In 7m mice, a significant preference for the target quadrant was found in WT mice (**E**), whereas Tg4-42 mice showed no clear preference for the target quadrant (**F**). Furthermore, 7m transgenic mice took significantly longer to reach the target than WT mice (**G**). At 12m only WT mice (**I**) showed a significant preference for the target quadrant, whereas Tg4-42 animals (**J**) showed no preference for the target quadrant. Time to reach the target did not differ in this age group (**K**). Swimming speed did not differ between WT and Tg4-42 mice at any age tested (**D**, **H**, **L**). *TQ* target quadrant, *LQ* left quadrant, *RQ* right quadrant, *OQ* opposite quadrant, *m* age in months. Quadrant preference: One-way repeated measures ANOVA followed by Bonferroni multiple comparisons; Time to reach the target and Swimming speed: unpaired *t*-test; n = 9–14. All Data presented as mean ± S.E.M. *p < 0.05, **p < 0.01, ***p < 0.001.

Three-month-old Tg4-42 and WT mice showed a significant preference for the target quadrant (Fig. 3A,B: one-way ANOVA followed by Bonferroni multiple comparisons, *quadrant preference:* **WT**: F(3,44) = 124.5, *p* < 0.001; Bonferroni for target vs. all other quadrants: *p* < 0.001, left vs. right quadrant: *p* = 0.0391; *quadrant preference:* **Tg4-42**: F(3,52) = 17.87, *p* < 0.001; Bonferroni for target vs. all other quadrants *p* < 0.001). In addition, the time to reach the target did not differ between transgenic and WT mice (Fig. 3C: unpaired *t*-test, *genotype:* F(11,13) = 7.992, *p* = 0.2284).

In contrast, 7 month-old Tg4-42 animals showed no clear preference for the target quadrant, whereas WT animals of the same age still showed a significant target quadrant preference (Fig. 3E,F: one-way ANOVA followed by Bonferroni multiple comparisons, *quadrant preference:* **WT**: F(3,48) = 26.98, *p* < 0.001; Bonferroni for target vs. all other quadrants: *p* < 0.001; *quadrant preference:* **Tg4-42**: F(3,40) = 2.993, *p* = 0.0421; Bonferroni for target vs. vs. right quadrant: *p* < 0.05). In addition, 7 month-old Tg4-42 animals took significantly longer to enter the target area than same-aged WT mice (Fig. 3G: unpaired *t*-test, *genotype*: F(10,12) = 11.08, 7 m p = 0.0043). The time to reach the target area did not differ between 12 month-old Tg4-42 and their same-aged WT controls (Fig. 3K: unpaired t-test, *genotype:* F(9,11) = 1.108, *p* = 0.2632). However, 12 month-old WT mice spent significantly more time in the target quadrant. In contrast, transgenic Tg4-42 mice showed no preference for the target quadrant indicating a robust deficit in spatial reference memory (Fig. 3I,J: one-way ANOVA followed by Bonferroni multiple comparisons, *quadrant preference:* **WT**: F(3,44) = 10.88, *p* < 0.001; Bonferroni for target vs. left quadrant: *p* < 0.05, target vs. right quadrant: *p* < 0.001, target vs. opposite quadrant: *p* < 0.01; *quadrant preference:* **Tg4-42**: F(3,32) = 4608, *p* = 0.0086; Bonferroni for opposite vs. target quadrant: *p* < 0.05). Swimming speed did not differ between the genotypes at all ages tested (Fig. 3D,H,L: unpaired *t*-test, *genotype:* **3m**: F(11,13) = 5.323, *p* = 0.2144; **7m**: F(10,12) = 5.451, *p* = 0.4186; **12m**: F(8,11) = 2.234, *p* = 0.2632).

In summary, the results of the acquisition and probe trial demonstrate age-dependent spatial learning and reference memory deficits in Tg4-42 mice.

### Search strategy analysis in acquisition training reveals early spatial navigation deficits in Tg4-42 mice

**Figure 4:**
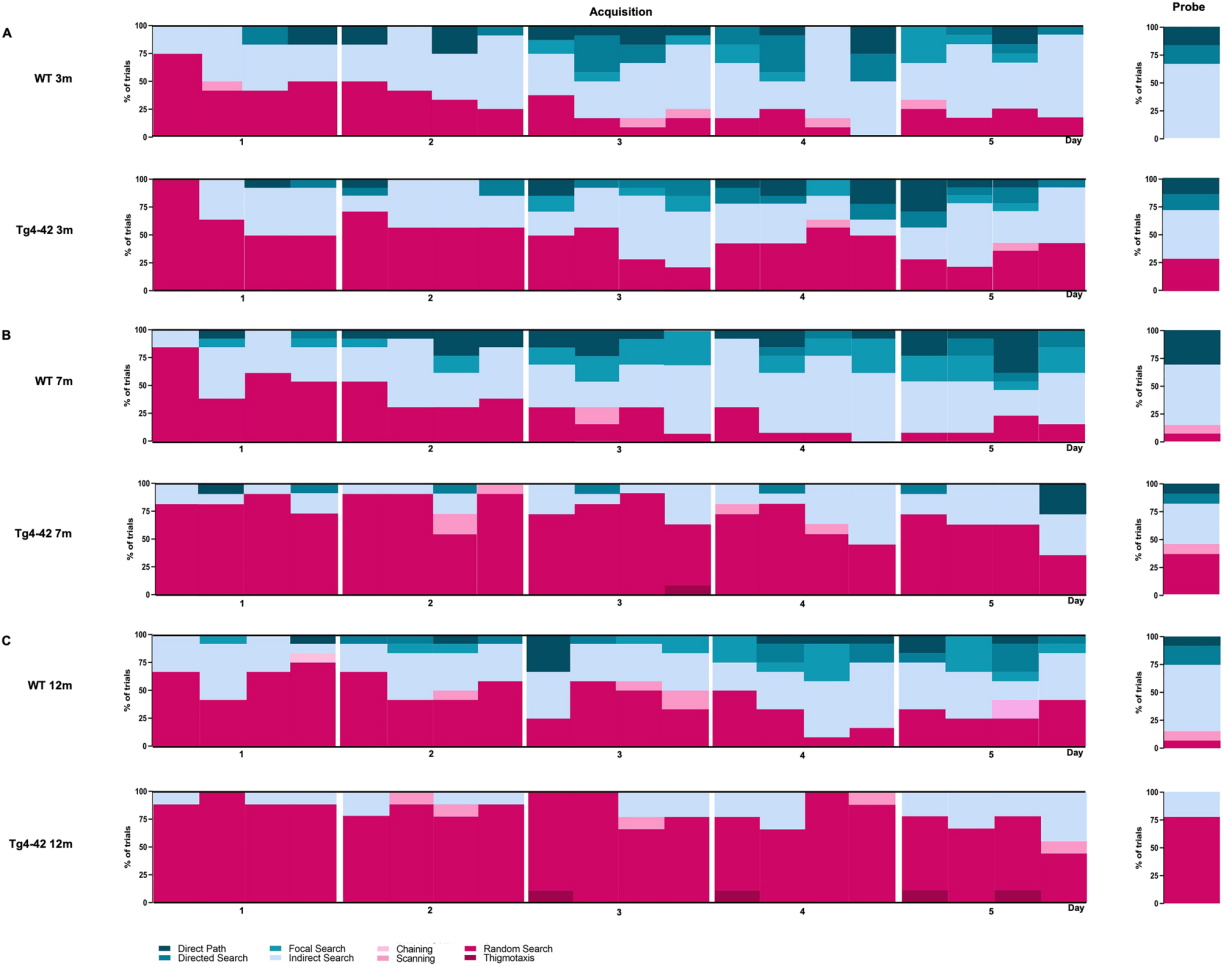
Qualitative analysis of spatial learning in Tg4-42 mice. Distribution of search strategies used by 3m (**A**), 7m (**B**) and 12m (**C**) old Tg4-42 and same-aged WT mice. WT animals showed a clear progression towards increasing spatial strategies over the 5 days of acquisition training. While non-spatial search strategies decreased in 3m Tg4-42 mice over the training days, Tg4-42 animals switched to spatial strategies significantly slower than WT animals. In contrast, 7m and 12m old Tg4-42 mice did not significantly change their search strategy pattern over the training days and mainly used non-spatial strategies. Data represents the percentage of search strategies performed in each trial over the 5 days of acquisition training and during the probe trial. n = 9–14.

During the first day of acquisition training, both female 3 month-old Tg4-42 and WT animals used predominantly a ‘random search’ strategy (Table S1, WT: 52%, Tg4-42: 66%; Fig. 4A: chi-square, *genotype:* **Day 1**: *p* = 0.4823). On the second day of training, WT animals switched to spatial search strategies using mainly ‘indirect search’ (50%). Search strategies differed significantly between WT and Tg4-2 mice on the second day of acquisition training (chi-square, *genotype:* **Day 2**: *p* = 0.0395), with Tg4-42 mice showing predominantly a ‘random search’ strategy (61%). As training progressed, non-spatial search strategies decreased in both WT and Tg4-42 mice. However, WT animals shifted more quickly to spatial strategies, as non-spatial strategies were almost absent by day 4 of acquisition training (12%). In contrast, Tg4-42 mice continued to show predominantly a ‘random search’ strategy (48%) until day 4 (chi-square, *genotype:* **Day 4**: *p* = 0.0043). During the last day of acquisition training the search strategies did not differ significantly between WT and transgenic mice (chi-square, *genotype:* **Day 5**: *p* = 0.2286) with both genotypes using a mixture of spatial search strategies. Overall 3 month-old Tg4-42 mice relied significantly more on non-spatial strategies than same-aged WT animals (Fig. 6: two-way repeated measures ANOVA followed by Bonferroni multiple comparisons, *genotype:* **3m**: *p* < 0.01).

**Figure 5:**
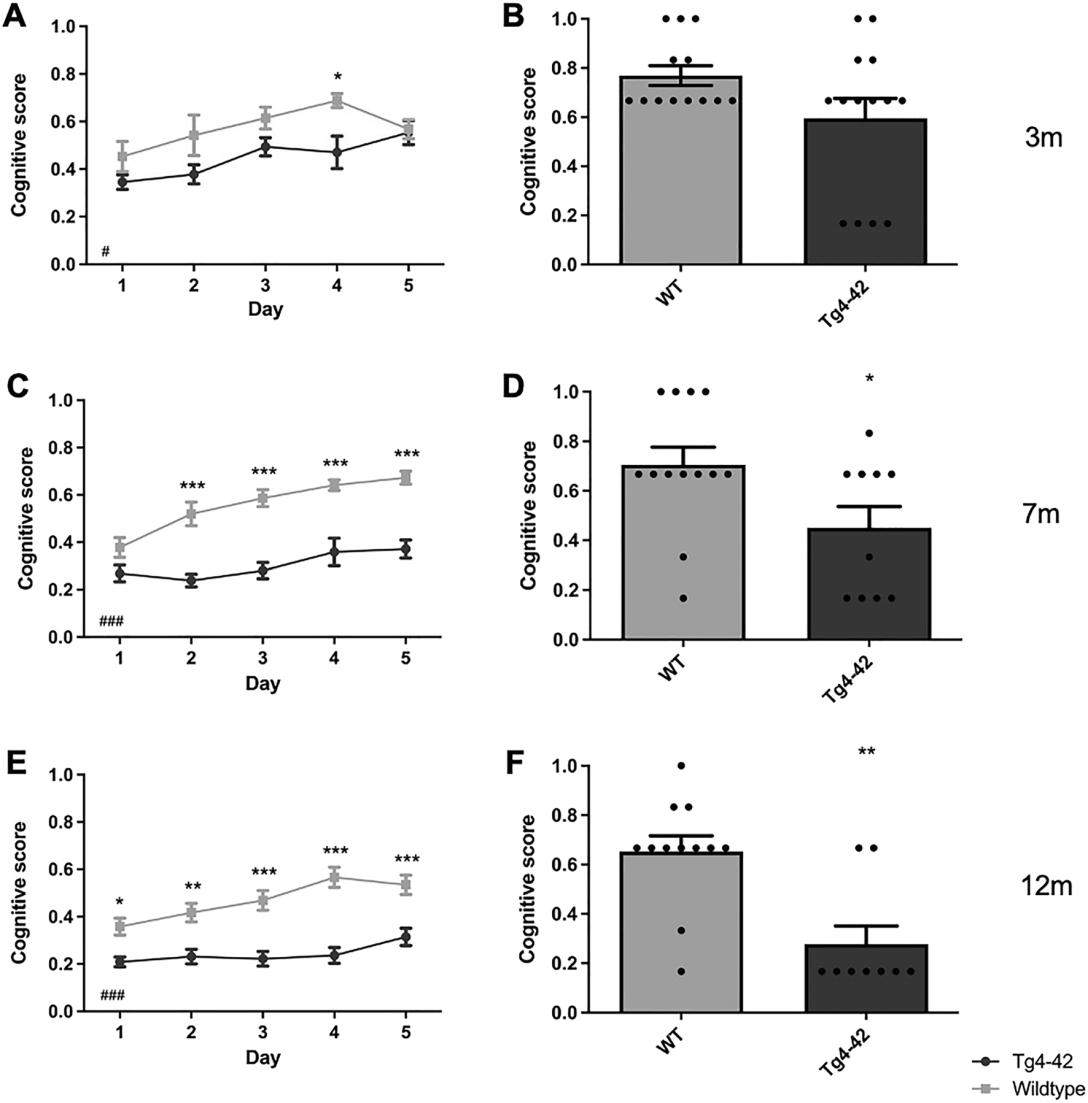
Cognitive Score is decreased in Tg4-42 mice. During the acquisition training of the MWM cognitive scores of female 3m (**A**), 7m (**C**) and 12m old Tg4-42 (**E**) were significantly lower than in same-aged WT animals. During the probe trial, 3m old Tg4-42 (**B**) mice show a similar cognitive score compared to WT mice. In contrast, 7m (**D**) and 12m old Tg4-42 (**F**) exhibit significantly reduced cognitive scores in the probe trial. Two-way repeated measures analysis of variance (ANOVA) followed by Bonferroni multiple comparisons. ANOVA: ^#^*p* < 0.05, ^###^*p* < 0.001; Bonferroni: *p < 0.05, **p < 0.01, ***p < 0.001. n = 9–14. Data presented as mean ± SEM.

The cognitive level of a particular search strategy can be quantified using a cognitive score that considers swim strategies based on their relevance to spatial learning. A higher cognitive score indicates primarily spatial learning, while non-spatial learning strategies such as ‘random search’, ‘scanning’ and ‘chaining’ result in a low cognitive score. The cognitive score of female 3 month-old Tg4-42 mice differed significantly from same-aged WT animals in the acquisition training (Fig. 5A: two-way repeated measures ANOVA, *genotype:* **3m**: F(1,24) = 6.438, *p* = 0.0181).

During the probe trial, 28% of 3 month-old Tg4-42 animals used a ‘random search’ strategy, whereas this strategy was not used by any WT mice, instead they relied solely on different forms of spatial search strategies. However, the cognitive score did not significantly differ between 3 month-old Tg4-42 and same-aged WT animals (Fig. 5B: unpaired t-test, *genotype:* **3m**: F(11,13) = 4.416, *p* = 0.0732).

**Figure 6:**
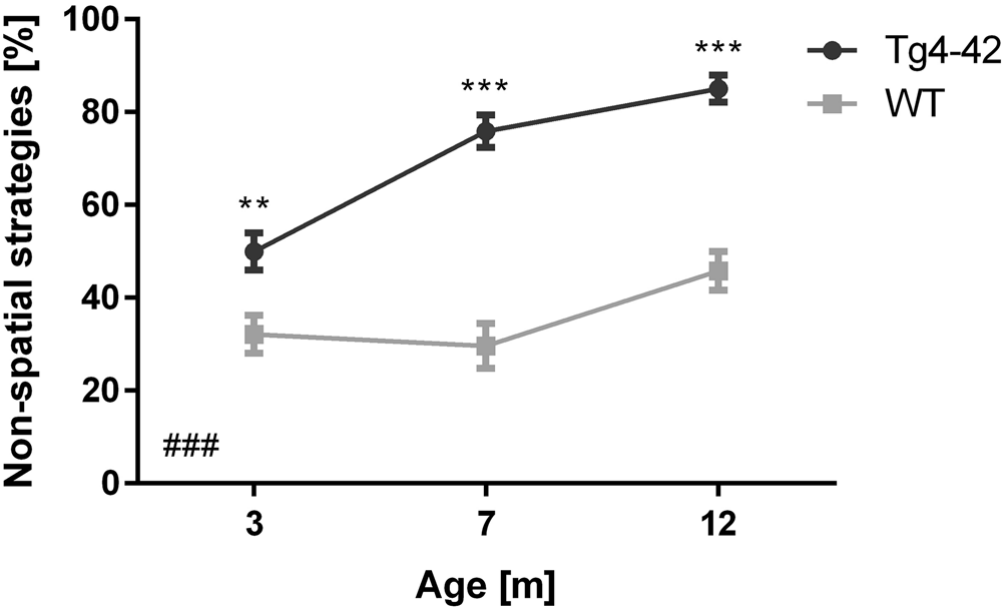
Tg4-42 mice use significantly more non-spatial search strategies than WT. Percentage of non-spatial search strategies for female Tg4-42 and WT mice during the acquisition training. Tg4-42 mice used significantly more non-spatial strategies than WT at any age tested. Two-way repeated measures ANOVA followed by Bonferroni multiple comparisons; n = 9–14. All Data presented as mean ± S.E.M. ANOVA: ^###^*p* < 0.001; Bonferroni: **p < 0.01, ***p < 0.001.

Female 7 month-old Tg4-42 mice and same-aged WT animals showed mainly ‘random search’ strategies (Table S1, WT: 60%, Tg4-42: 82%) during the first day of acquisition training and the overall applied search strategies did not differ between the genotypes (Fig. 4B: chi-square, *genotype:* **Day 1**: *p* = 0.1350). Over the subsequent days, the fraction of ‘random search’ declined and WT mice used spatial strategies nearly exclusively. During the last day of acquisition training, 7 month-old WT animals used in 87% of the trials a spatial strategy to locate the platform with ‘indirect search’ (40%), ‘focal search’ (19%), and ‘direct path’ (17%) becoming the most dominant search strategies. In contrast, the performance of female Tg4-42 did not improve over the 5 days of training and the search strategies to locate the hidden platform were significantly different from WT animals (chi-square, *genotype*: **Day 2**: *p* < 0.001; **Day 3**: *p* < 0.001; **Day 4**: *p* < 0.001; **Day 5**: *p* < 0.001). Transgenic mice used predominantly non-spatial search strategies to find the platform location and during the last day of acquisition training, Tg4-42 still employed a ‘random search’ strategy in 60% of the trials and ‘scanning’ in 5% of the trials. Overall 7 month-old Tg4-42 mice relied significantly more on non-spatial strategies than WT animals (Fig. 6: two-way repeated measures ANOVA followed by Bonferroni multiple comparisons, *genotype:* **7m**: *p* < 0.001). In line with these findings, female 7-month-old transgenic mice showed a lower cognitive score than same-aged WT mice in the acquisition training (Fig. 5C: two-way repeated measures ANOVA, *genotype*: **7m**: F(1,22) = 81.98, *p* < 0.001).

During the probe trial 45% of Tg4-42 mice used non-spatial search strategies with predominantly ‘random search’, while only 15% of WT animals used non-spatial strategies. In addition, 7 month-old WT animals showed a significantly higher cognitive score compared to same-aged Tg4-42 mice (Fig. 5D: *t*-test, *genotype*: F(12,10) = 2.304, *p* = 0.0316).

During the first day of acquisition training, female 12-month-old Tg4-42 and WT animals used predominantly a ‘random search’ strategy (Table S1, WT: 63%, Tg4-42: 92%) to locate the hidden platform. However, the overall search pattern differed significantly between the genotypes as WT mice already used a spatial search strategy in approximately 35% of the trials (Fig. 4C: chi-square, *genotype*: **Day 1**: *p* = 0.0472). Over the course of the acquisition training, the proportion of ‘random searches’ of WT mice decreased and the number of spatial strategies increased from 35 on day 1 to 65% on day 5. In contrast, Tg4-42 were not able to develop and use spatial strategies during their navigation. Instead, they adopted a non-spatial ‘random search’ strategy that predominated on all days (Day 2: 83%; Day 3: 83%, Day 4: 80%, Day 5: 61%). The search strategies used by 12 month-old female Tg4-42 animals differed significantly from WT on all days of acquisition training (Fig. 4C: chi-square, *genotype*: **Day 1**: *p* = 0.0472; **Day 2**: *p* = 0.0282; **Day 3**: *p* = 0.0047; **Day 4**: *p* < 0.001; **Day 5**: *p* = 0.0073). Overall Tg4-42 mice relied significantly more on non-spatial strategies than WT animals (Fig. 6: two-way repeated measures ANOVA followed by Bonferroni multiple comparisons, *genotype:* **12m**: *p* < 0.001). Consistent with these results, the cognitive score was significantly different between female 12 month-old Tg4-42 and WT mice in the acquisition training (Fig. 5E, two-way repeated measures ANOVA, *genotype*: **12m**: F(1,19) = 45.34, *p* < 0.001). The cognitive score did not differ between 3, 7 and 12 month-old WT mice (two-way repeated measures ANOVA, **WT** *age*: F(2,34) = 4.478, *p* = 0.4602). In contrast, Tg4-42 showed a decreased cognitive score with age (two-way repeated measures ANOVA, **Tg4-42** *age*: F(2,31) = 17.71, *p* < 0.001).

During the probe trial 78% of 12-month-old Tg4-42 mice used a ‘random search’ strategy. In contrast, 83% of WT animals used spatial search strategies with predominantly ‘indirect search’. Furthermore, WT animals demonstrated a significantly higher cognitive score than same-aged Tg4-42 mice (Fig. 5F: *t*-test, *genotype:* F(8,11) = 3.877, *p* = 0.0010).

### Decreased metabolic activity in the hippocampus of Tg4-42 mice

**Figure 7:**
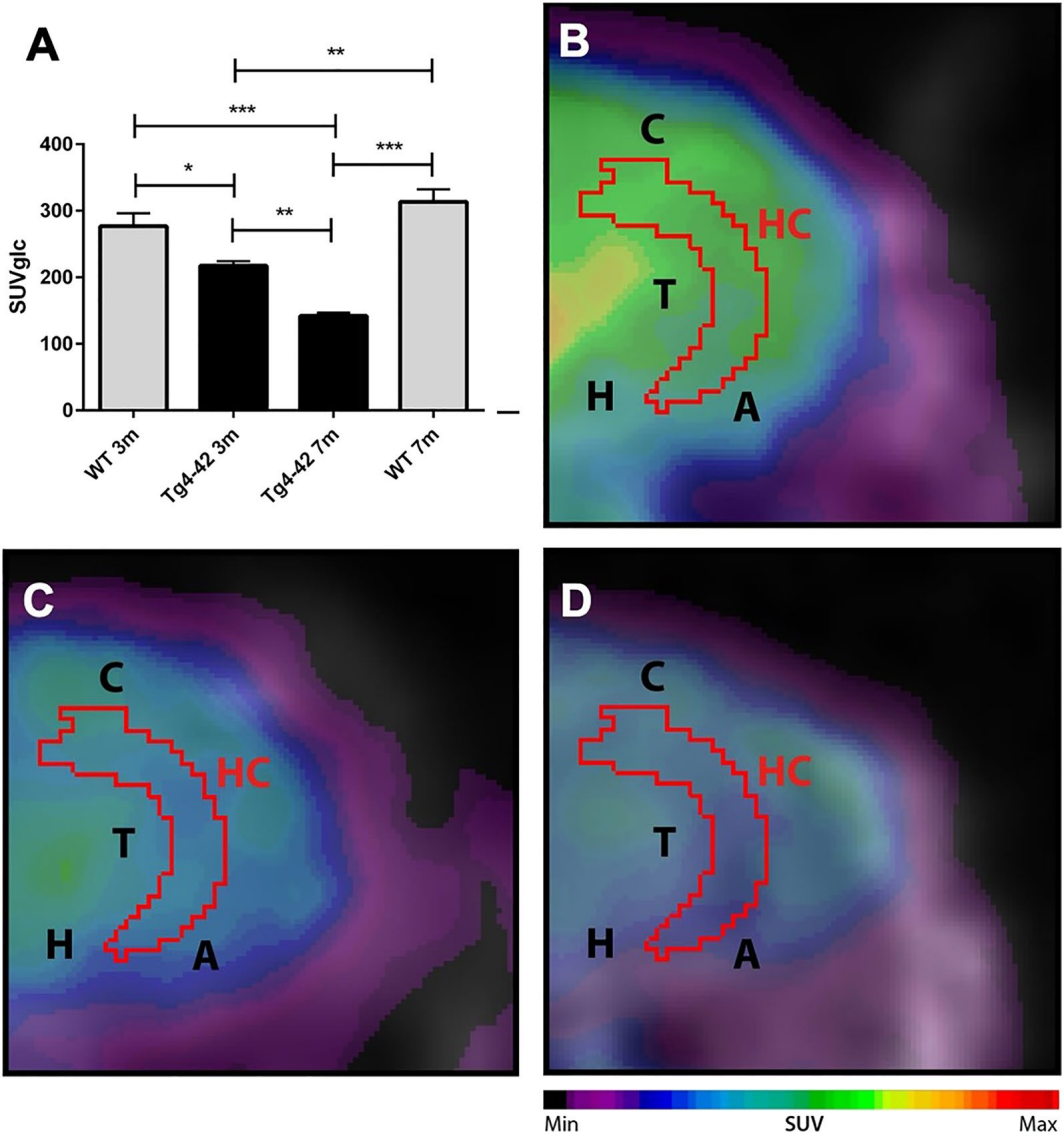
^18^F-FDG-PET shows decreased metabolic activity in the hippocampus of Tg4-42 mice. (**A**) Quantification of ^18^F-FDG uptake in the hippocampus. ^18^F-FDG-uptake in the hippocampus was significantly reduced in 3m and 7m Tg4-42 mice compared to same-aged WT animals. Hypometabolism in Tg4-42 increases age-dependently. (**B**) Fused ^18^F-FDG-PET/MRI of a WT mouse in coronal view. (**C**) Fused ^18^F-FDG-PET/MRI of a 3-month-old Tg4-42 mouse in coronal view with distinctly lower FDG uptake compared to the WT mouse. (**D**) Fused ^18^F-FDG-PET/MRI of a 7-month-old Tg4-42 mouse in coronal view with distinctly lower FDG uptake compared to WT and 3-month-old Tg4-42 mice. One-way-ANOVA; ***p < 0.001; **p < 0.01; *p < 0.05; *WT* wild-type; *m* months. *A* Amygdala, *C* Cortex, *H* Hypothalamus, *Hc* Hippocampus, *T* Thalamus.

^18^F-FDG-PET/MRI was used to determine hippocampal glucose metabolism. Quantitative analysis of FDG-uptake was performed using a mouse brain atlas and blood glucose corrected SUV values (SUVglc) were measured within a predefined cerebellum VOI (Fig. 7). Three-and 7-month-old Tg4-42 and mice showed significantly decreased ^18^F-FDG uptake in the hippocampus compared to WT mice (Fig. 7A, one-way ANOVA: *genotype:* F(3,18) = 22.9, *p* < 0.001; Bonferroni multiple comparisons: 3 WT vs. 3 m Tg4-42: *p* < 0.05; 3 m WT and 7 m WT vs. 7 m Tg4-42: *p* < 0.001; 3 m Tg4-42 vs. 7 m Tg4-42 *p* < 0.01; 7 m WT vs. 3 m Tg4-42 *p* < 0.01).

## Discussion

The present study demonstrates that female Tg4-42 mice have an age-dependent impaired spatial reference memory. In line with previous findings^15,20,21^, conventional behavior analysis of the escape latencies and quadrant preference, confirmed spatial memory and spatial reference memory deficits in female 7 and 12 month-old Tg4-42 mice. In contrast, conventional analysis of the MWM indicated that spatial memory is intact in 3 month-old Tg4-42 mice. However, by studying navigation strategies early spatial navigation deficits could already be detected in young Tg4-42 mice.

Assessing spatial memory using escape latencies provides a global picture of memory performance, but not a detailed picture of how an animal with developing pathology solves a spatial task. Therefore, a detailed analysis of the swimming strategies was performed to better describe the behavioral differences between Tg4-42 and WT mice. Tg4-42 mice, regardless of age, used a qualitatively and quantitatively different search strategy pattern than WT animals. In general, Tg4-42 mice used more non-spatial strategies and fewer spatial strategies. WT animals, independent of age, as well as 3-month-old Tg4-42 mice, changed their search strategies towards more spatial strategies over the training days and these changes were likely the primary cause of improvement during the acquisition phase.

The predominantly use of ‘random search’ strategy in 7 and 12 month-old Tg4-42 mice resulted in virtually no improvement in their escape latency over the 5 days of acquisition training. In contrast, same-aged WT animals quickly adopted spatial search strategies and rapidly reduced their escape latencies during training. Similar behavior has been described in TgCRND8 mice. Janus^25^ demonstrated that 6-month-old TgCRND8 display spatial working memory deficits while relying mostly on non-spatial, chaining strategies to locate the hidden platform in the MWM^25^. Similarly, deficits in PDAPP mice could be explained by the use of non-spatial strategies and especially repetitive looping strategies^26^. In addition, Karunakaran showed that preferential use of the noncognitive search strategy circling resulted in an increased number of unsuccessful trials during MWM learning phase in 2-month-old male APP/PS1 mice^27^.

Analysis of search strategies revealed early behavioral changes in 3 month-old Tg4-42 that otherwise would have been overlooked if we had focused only on escape latencies. Young Tg4-42 held on to non-spatial strategies longer than WT mice during the acquisition training. While non-spatial strategies were almost absent in WT animals by day four of acquisition training Tg4-42 mice still used mainly non-spatial search strategies to locate the platform. Furthermore, one-third of 3 month-old Tg4-42 relied on a ‘random search’ strategy during the probe trial whereas WT mice solely resorted on spatial strategies. In contrast, classic analysis of swimming distribution in the probe trial failed to detect these early changes in Tg4-42 mice. Therefore, we could show for the first time early spatial navigation deficits in Tg4-42 at 3 months. These deficits occur before the onset of severe neuron loss^15,20^. However, the detected deficits are in good agreement with the here observed hippocampal hypometabolism and synaptic alterations in the hippocampus of Tg4-42 mice^22^.

The hippocampus plays a crucial role in spatial navigation and analyzing search strategies of mice during the MWM task allows to differentiate between hippocampus-dependent allocentric and hippocampus-independent egocentric search strategies^28–30^. Egocentric navigation is independent of environmental cues and relies on directional responses (left–right) and remembering locations of objects with respect to self. On the other hand, allocentric navigation is based on external cues and allows navigating from different starting points to a target as long as the external landmarks remain the same^29,31^. These hippocampus-dependent and spatial search strategies include ‘direct path’, ‘directed search’, ‘focal search’, and ‘indirect search’. As acquisition training progresses, an increasing contribution of allocentric knowledge leads to improved accuracy in finding the hidden platform as seen in WT animals in this study. It has been shown that animals with lesions in the hippocampus fail to form an allocentric cognitive map and need to rely on egocentric strategies^32–36^. This is well in line with the current study as aged Tg4-42 mice rely mainly on egocentric strategies while displaying a severe neuron loss in the hippocampus. It has been shown that 7-month-old Tg4-42 animals display a neuron loss of more than 50% in the CA1 region of the hippocampus as well as a 28% neuron loss in the CA2/3 region and 14% loss in the dentate gyrus^19,20^. In addition to the hippocampus, other brain structures are known to affect spatial memory by influencing motion organization, spatial navigation, or motor performance. These structures include the amygdala, prefontal cortex, striatum, cerebellum, thalamic structures, and locus coeruleus^37,38^. Furthermore, other brain regions that are involved in allocentric search navigation include the perirhinal cortex, parietal cortex, and entorhinal cortex^39–41^.

Importantly, egocentric and non-spatial based search strategies that are often used by animals with hippocampal lesions can also be efficient to a certain degree. Although non-spatial strategies such as ‘random search’ and ‘scanning’ can be successful and result in lower escape latencies, these strategies are not indicators of spatial memory. Eichenbaum et al.^28^ showed that rats with lesions in the hippocampus displayed decreased escape latencies and path lengths in the MWM. However, these mice only remembered the distance of the target platform from the wall and found the platform by circling the pool. Wiesmann et al.^42^ showed that APP/PS1 mice on a multi-nutrient diet improved spatial learning by changing their search pattern from ‘random search’ and ‘scanning’ to ‘chaining’. The authors pointed out that APP/PS1 mice developed a highly efficient way to locate the goal platform that is not dependent on an intact hippocampus^42^. Consistent with these observations, 3-month-old Tg4-42 mice displayed escape latencies similar to same-aged WT animals, although they used significantly fewer spatial strategies and relied more on non-spatial strategies during the acquisition training.

In AD patients topographical disorientation, the inability to orient oneself in one's surroundings, is an early symptom of AD and often causes patients to wander and get lost^43^. Thereby, allocentric deficits could be detected in MCI and preclinical AD^44,45^. As allocentric-specific deficits have been detected in asymptomatic preclinical AD allocentric spatial memory tasks may be useful in the early diagnosis of AD^1,4^. Similarly, the present study demonstrates for the first time early allocentric-specific deficits in 3 month-old Tg4-42 mice before the onset of severe memory deficits. Therefore, swimming strategies may have practical implications when testing potential AD therapeutics and could serve as an early therapeutic outcome measure.

Importantly, the impaired spatial working memory in aged Tg4-42 mice cannot explained by a consistent thigmotaxic swimming behavior. Thigmotaxis is an often described behavior pattern in transgenic mice and refers to an animal's tendency to remain close to the walls of its environment^46^. In the MWM a thigmotaxic behavior is associated with longer escape latencies and therefore mice have difficulty finding the location of the platform^47,48^. As a result, spatial learning ability cannot be adequately assessed. However, the described impairments of Tg4-42 mice cannot be attributed to an increased thigmotaxis as transgenic mice did not differ from WT animals in thigmotaxic swims, floating rate, or swimming speed. Furthermore, the described impairments of Tg4-42 mice are not a result of previously reported motor deficits in Tg4-42 mice^24^. While motor impairments motor can impair cognitive performance in the MWM, we could rule motor deficits as a confounding factor on the cognitive parameters in the MWM as swimming speed did not differ between Tg4-42 and WT mice at any age tested. This is in line with Wagner et al.^24^ who showed that balance and motor coordination in aged Tg4-42 mice is impaired, while general locomotor activity and muscle strength is not altered. Furthermore, it has been shown that impairments of MWM learning are independent of locomotor effects, as reductions in locomotor land speed does not necessary affect swimming speed^11^. In addition, it should be noted that 12 month-old Tg4-42 mice performed significantly worse than same-aged WT animals during the cued training, so that visual impairments cannot be excluded. As there is a possibility that sensory and cognitive deficits during aging are confounded in these mice, additional nonvisual test should be performed in the future^49^. However, 12 month-old Tg4-42 significantly improved over the 3 days of cued training and did not perform different from WT animals on the last day of training.

Several groups have described manual and automated methods for classifying search strategies in MWM in recent years and have demonstrated the utility of such an additional analysis tool^50–54^. Previous search strategy analysis revealed that spatial accurate search is impaired in animal models of autism, aging, traumatic brain injury as well as AD^25,26,55,56^. Furthermore, search strategy analysis demonstrated that spatially precise search is promoted by adult neurogenesis and the involvement of the ventral hippocampus in coarse spatial goal-orientated search and cue-dependent navigation^57,58^. In addition, Harvey et al.^59^ examined the searching strategies in relation to the number of distal cues. Despite these applications and findings, search strategy analysis is still not routinely used to study the learning ability and performance of mice in the MWM. This is likely because commercially available software packages often do not provide these analyses, and the analytical methods used in previous work are often not available in the form of an easy-to-use software package.

We recognize that our study has limitations. Although the current work extends previous MWM studies by focusing on search strategies next to escape latencies, it has to be noted, that the Pathfinder analysis only indicates which search strategy is predominantly used but fails to notice transitions between different strategies within a single trial. However, compared to a human observer, Pathfinder allows a strict objectivity in the classification of search strategies.

Furthermore, the study was only performed on female mice. Although the reason is not yet known, AD has a gender-specific epidemiologic profile, with women disproportionately affected in both prevalence and severity^60,61^. A number of female transgenic AD mouse models also display an earlier onset of AD pathologies as well as a more severe pathology than their male counterparts^62–64^. Moreover, previous studies demonstrated sex differences in various AD mouse models, while behavior deficits are often more severe in female mice^65–70^. Interestingly, in young APP/PS1 search strategy alterations could only been detected in male mice, while female APP/PS1 demonstrated the same search pattern as WT animals^27^. Therefore, future studies should also analyze the search strategy of male Tg4-42 mice in the MWM.

A number of female transgenic AD mouse models also display an earlier onset of AD pathologies as well as a more severe pathology than their male counterparts (40–42). Moreover, behavior deficits are often more severe in female mice.

In summary, simply quantifying escape latency or swimming distance can lead to a partial or inaccurate understanding of the behavior and cognitive abilities of mice. The present study is the first to show impairments in spatial navigation in 3-month-old Tg4-42 mice. Furthermore, we could demonstrate that the spatial reference memory deficits in aged Tg4-42 animals are caused by the lack of allocentric and spatial strategies. Therefore, analyzing search strategies in the Tg4-42 model can be a powerful tool for preclinical drug testing and identifying early or subtle therapeutic successes.

## Material and methods

### Tg4-42 transgenic Mice

The generation of the Tg4-42 mouse line has been previously described^15^ and is available through Prof. Thomas A. Bayer. In brief, Tg4-42 mice express human Aβ4-42 fused to the murine thyrotropin-releasing hormone (TRH) signal peptide under the control of the neuronal Thy-1 promoter. Tg4-42 mice were generated and maintained on a C57Bl/6 J genetic background (Jackson Laboratories, Bar Harbor, ME, USA). In this study, female 3, 7 and 12 month-old homozygous Tg4-42 mice and wildtype (WT) control C57Bl/6 J mice were used (WT: 3 m n = 12; 7 m n = 13; 12 m n = 12; Tg4-42: 3 m n = 14; 7 m n = 11; 12 m n = 9). Each individual mouse was tested at one age point only. As Tg4-42 mice were bred using homozygous breeders, no non-transgenic control mice could be received from this breeding. To ensure that the genetic background of study groups does not influence the animal’s phenotype, a control study was performed comparing the non-transgenic control mice with a C57BL6 background with female non-transgenic littermates of a hemizygous breeding at the age of 3 months. This analysis resulted in no significant differences between the two non-transgenic control groups (Fig. S2). Mice were housed in individually ventilated cages in a controlled environment on a 12/12 h light/dark cycle in groups up to five (randomly divided with 10 weeks). Water and food were available ad libitum. All animals were handled according to the German guidelines and EU legislations for animal care and the experiments were approved by the local authorities (Niedersächsisches Landesamt für Verbraucherschutz und Lebensmittelsicherheit, Röverskamp 5, 26203 Oldenburg, Germany, [15/1760] and Landesamt für Gesundheit und Soziales LAGeSo Darwinstr. 15, 10589 Berlin [65/18, 260/19]). All experiments followed the recommendations in the ARRIVE guidelines, and experimenters were blinded to the genetic status of the mice.

### Morris water maze

To evaluate spatial reference memory, the Morris Water Maze (MWM) was performed as previously described^10,15^. Therefore, mice were trained to search a hidden circular platform (10 cm) in a pool (110 cm diameter) filled with opaque water by using different cues. The pool was filled with tap water mixed with non-toxic white paint. During the whole test the water temperature was maintained at 20 ± 2 °C. The pool was divided into four virtual quadrants based on the platform localization: left, right, opposite, and target.

Each mouse went through 3 days of cued training, 5 days of acquisition training and a final probe trial. During the cued training the platform was marked with a triangular flag. During the cued training no additional distal cues were present. Mice were introduced into the water near the edge of the pool facing the wall. They were given one minute to find the submerged platform. The platform was located in the upper third of the quadrant (14 cm from the rim of the pool). If they failed to find the platform in one minute, they were gently guided to it. Every mouse had to sit on the platform for 10 s before being removed from the pool. To prevent hypothermia mice were kept in front of a heat lamp until they were dry. The cued training consisted of four trials per day with an average inter-trial interval of 15 min. The start position and the position of the platform changed for every trial.

Forty-eight hours after the last cued training day, 5 days of acquisition training started. For the acquisition training the flag was removed from the platform. In addition to the distal cues in the room, proximal visual cues were attached to the edge of the pool. During the acquisition training, mice were placed in the water from one of four predefined entry points, while the location of the platform remained stationary. Trials were performed as during the cued training phase.

Twenty-four hours after the last acquisition trial, a probe trial was performed to assess spatial reference memory. For the probe trial the platform was removed from the pool, and mice were introduced into the water from a novel entry point (opposite of the former platform location). Mice were allowed to swim freely for one minute. To calculate the time to reach the target, the position of the platform in the tracking software was set to the same position as in the previous acquisition training. For tracking the escape latency, quadrant preference and swimming speed, ANY-Maze video tracking software (Stoelting Co., Wood Dale, USA) was used.

### Search strategy analysis

Searching strategies during the acquisition training and probe trial were analyzed with Pathfinder (Jason Snyder Lab, Vancouver, Canada)^14^. Eight possible swim strategies were differentiated (Fig. 1): ‘direct path’ (Ideal path error [IPE] ≤ 1250 mm; Heading error ≤ 40°), ‘directed search’ (time in angular corridor ≤ 70% of trial; distance covered ≤ 4000 mm; IPE ≤ 15,000 mm), ‘focal search’ (distance to swim path centroid ≤ 30% of radius; distance to goal ≤ 30% of radius; distance covered ≥ 1000 mm and ≤ 4000 mm), ‘indirect search’ (IPE ≤ 3000 mm; average heading error ≤ 360°), ‘chaining’ (time in annulus zone ≥ 90% of trial; quadrants visited ≥ 4; area of maze traversed ≤ 40% of maze), ‘scanning’ (area of maze traversed ≥ 5% and ≤ 20% of maze; average distance to maze center ≤ 60% of radius), ‘random search’ (area of maze traversed ≥ 10% of maze) and ‘thigmotaxis’ (time in full thigmotaxis zone ≥ 65% of trial; time in smaller thigmotaxis zone ≥ 35% of trial; total distance covered ≥ 4000 mm). The different spatial parameters were adjusted to the experimental setup (goal position [x/y]: 275, 775; goal diameter: 200; maze diameter: 1100; maze center [x/y]: 550, 550; angular corridor width: 40; chaining annulus width: 200; thigmotaxis zone size: 50). Spatial strategies included ‘direct path’, ‘directed search’, ‘focal search’ and ‘indirect search’. ‘Chaining’, ‘scanning’, ‘random search’ and ‘thigmotaxis’ were considered as non-spatial strategies. Thereby, ‘direct path’ is defined as a nearly perfect trajectory to the platform with minimal deviation from a straight path. ’Directed search’ describes a swim strategy with slight deviation from a direct path. ‘Focal search’ refers to a spatially restricted search (in the center portion of the pool), while ‘indirect search’ indicates a spatially targeted search that contains a major directional error. Furthermore, ‘chaining’ describes a spatially non-specific strategy were mice search in a fixed distance from pool wall. ‘Scanning’ refers to a random strategy that avoids the wall. ‘Random search’ does not include a spatial search pattern and ‘thigmotaxis’ indicates a swim path limited to the pool wall^14^.

### Cognitive score

Cognitive performance was evaluated using a scoring system^54^ in which higher cognitive strategies received higher scores: thigmotaxis = 0; random search = 1; scanning = 2, chaining = 3; indirect search = 4; focal search = 4; directed search = 5; direct path = 6. The average cognitive score was calculated for each mouse per day and normalized to six, the highest possible score.

### ^18^F-FDG PET/MRI

^18^F-fluoro-deoxy-glucose positron emission tomography/magnetic resonance image (^18^F-FDG-PET/MRI) acquisition and analysis were used to evaluate brain glucose metabolism in the hippocampus of 3 and 7 month-old Tg4-42 mice (n = 4–5) as previously described^71^. Imaging was performed 8 ± 2 days after the probe trial. Mice were fasted overnight and blood glucose levels were measured. In brief, 14–19 MBq (mean 17.19 MBq) of ^18^F-FDG were administered intravenously into a tail vein with a maximum volume of 200 μl. Mice were anesthetized with isoflurane supplemented with oxygen during the scans and were awake during the uptake period. After an uptake period of 45 min, PET imaging was performed on a small animal 1 Tesla nanoScan PET/MRI (Mediso, Hungary). During the scan mice were placed on a 37 °C heated bed. Respiration rate was monitored constantly during the scans. PET scans were performed for 20 min. MRI-based attenuation correction was conducted (matrix 144 × 144 × 163 with a voxel size of 0.5 × 0.5 × 0.6 mm3, TR: 15 ms, TE 2.032 ms and a flip angle of 25°) and the PET images were reconstructed with the following parameters: matrix 136 × 131 × 315 with a voxel size of 0.23 × 0.3 × 0.3 mm3. Image analysis was performed using PMOD v3.9 (PMOD Technologies, Switzerland). A predefined mouse brain atlas template was used to analyze different brain areas including the hippocampus. Corresponding PET images were matched to the MRI and statistics within the hippocampus volume of interest (VOI) in kBq/cc were generated. Standardized uptake value (SUV) was calculated [SUV = tissue activity concentration average (KBq/cc) × body/weight (g)/injected dose (kBq)] for semi-quantitative analysis and SUV values were corrected for blood glucose levels [SUVGlc = SUV × blood glucose level (mg/dl)].

### Statistical analysis

Differences between groups were tested with unpaired t-test, one-way analysis of variance (ANOVA) followed by Bonferroni multiple comparison or two-way analysis of variance (ANOVA) followed by Bonferroni multiple comparisons as indicated. For comparison of the eight possible search strategies between groups chi-square analysis was performed. Significance levels were defined as follows: ***p < 0.001, **p < 0.01, *p < 0.05. All data were analyzed using GraphPad Prism 9.1.2 (GraphPad Software, San Diego, CA, USA).

## Supplementary Information

Supplementary Information.
