# Supplementary material for: Search strategy analysis of Tg4-42 Alzheimer Mice in the Morris Water Maze reveals early spatial navigation deficits
Source: Sci Rep. 2022 Mar 31;12:5451. doi: 10.1038/s41598-022-09270-1 (PMC8971530; doi:10.1038/s41598-022-09270-1)

Table S1 Percentage of search strategies used during the Acquisition training

Strategy	Day 1		Day 2		Day 3		Day 4		Day 5	
3m female	**WT**	**Tg4-42**	**WT**	**Tg4-42**	**WT**	**Tg4-42**	**WT**	**Tg4-42**	**WT**	**Tg4-42**
Direct Path	4.17	1.78	8.33	1.79	10.42	3.57	8.33	10.71	4.17	10.71
Directed Search	4.17	1.78	2.10	5.36	12.50	7.14	18.75	8.93	6.25	10.71
Focal Search	0.00	0.00	0.00	0.00	6.25	8.93	6.25	3.57	12.50	3.57
Indirect Search	37.5	30.36	50	32.14	41.67	41.07	52.08	26.79	54.17	41.07
Chaining	0.00	0.00	0.00	0.00	0.00	0.00	0.00	0.00	0.00	0.00
Scanning	2.10	0.00	0.00	0.00	4.17	0.00	2.08	1.79	2.08	1.79
Random Search	52.10	66.07	39.58	60.71	25	39.29	12.5	48.21	20.83	32.14
Thigmotaxis	0.00	0.00	0.00	0.00	0.00	0.00	0.00	0.00	0.00	0.00
7m female	**WT**	**Tg4-42**	**WT**	**Tg4-42**	**WT**	**Tg4-42**	**WT**	**Tg4-42**	**WT**	**Tg4-42**
Direct Path	1.92	2.27	13.46	0.00	11.54	0.00	7.69	0.00	17.31	0.00
Directed Search	1.92	2.27	0.00	2.27	0.00	2.27	3.85	2.27	9.62	9.09
Focal Search	3.85	0.00	5.77	0.00	23.08	0.00	15.38	0.00	19.23	0.00
Indirect Search	32.69	13.64	42.31	9.09	40.38	20.45	61.54	29.55	40.38	27.27
Chaining	0.00	0.00	0.00	0.00	0.00	0.00	0.00	0.00	0.00	0.00
Scanning	0.00	0.00	0.00	6.82	3.85	0.00	0.00	4.55	0.00	4.55
Random Search	59.62	81.82	38.46	81.82	21.15	75.00	11.54	63.64	13.46	59.09
Thigmotaxis	0.00	0.00	0.00	0.00	0.00	2.27	0.00	0.00	0.00	0.00
12m female	**WT**	**Tg4-42**	**WT**	**Tg4-42**	**WT**	**Tg4-42**	**WT**	**Tg4-42**	**WT**	**Tg4-42**
Direct Path	2.08	0.00	2.08	0.00	8.33	0.00	6.25	0.00	6.25	0.00
Directed Search	2.08	0.00	6.25	0.00	2.08	0.00	8.33	0.00	10.42	0.00
Focal Search	0.00	0.00	4.17	0.00	6.25	0.00	16.67	0.00	12.50	0.00
Indirect Search	31.25	8.33	33.33	11.11	35.42	11.11	41.67	14.29	35.42	30.56
Chaining	2.08	0.00	0.00	0.00	0.00	0.00	0.00	0.00	0.00	0.00
Scanning	0.00	0.00	2.08	5.56	6.25	2.78	0.00	2.86	4.17	2.78
Random Search	62.50	91.67	52.08	83.33	41.67	83.33	27.08	80.00	31.25	61.11
Thigmotaxis	0.00	0.00	0.00	0.00	0.00	2.78	0.00	2.86	0.00	5.56

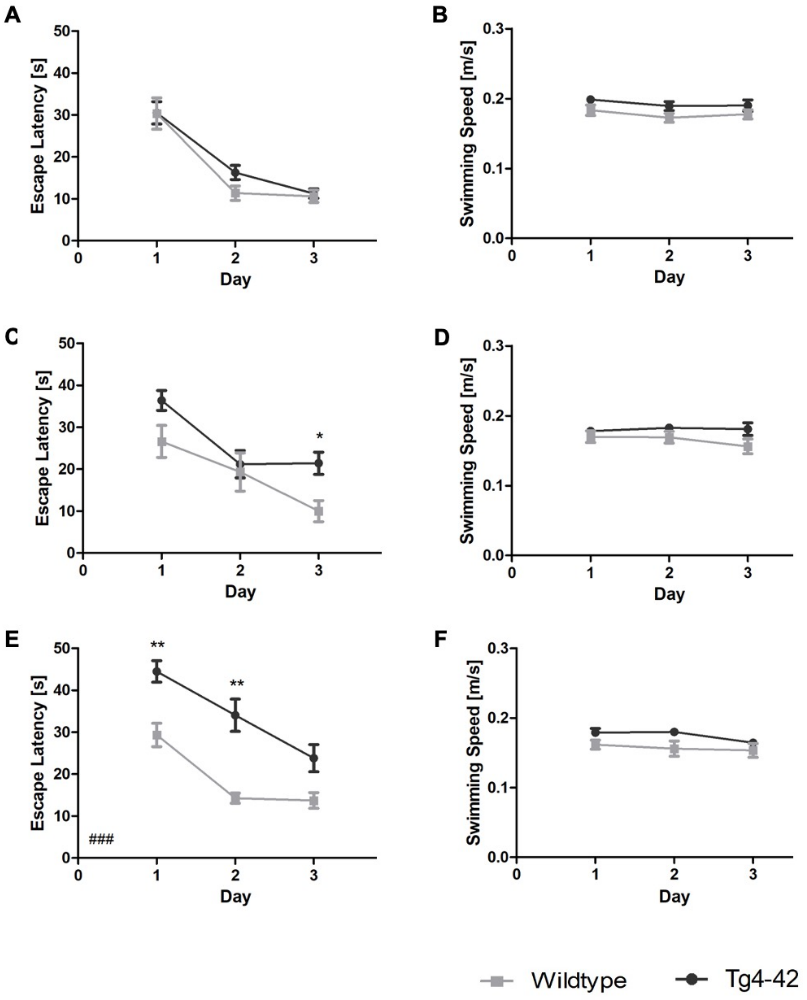

Figure S1 Cued training indicates the motoric ability and intact vision to perform the MWM. Homozygous Tg4-42- and WT mice were tested at 3m (A-B), 7m (C-D) and 12m (E-F). In each age group, escape latencies (A, C, E) decreased significantly over the course of the test. Swimming speed (B, D, F), which served as a control, did not differ between genotypes and training days. Two-way repeated measures ANOVA followed by Bonferroni multiple comparisons; n=9-14. All Data presented as mean ± S.E.M. ANOVA: $^{###}p<0.001$; Bonferroni: $^{*}p<0.05$, $^{**}p<0.01$.

Figure S2 WT animals from a Tg4-42 breeding (WT new) did not perform significant different from WT animals from a non Tg4-42 breeding (WT) (A) During the Cued training WT and WTnew showed comparable escape latencies F(1,18)=2.806, p=0.112. (B) Swimming speed did not differ between WT and WTnew in the cued training F(1,18)=1.485, p=0.287. (C) Escape latencies in the acquisition training did not differ between WT and WTnew F(1,18)=0.4226, p=0.5239. (D) Swimming speed did not differ between WT and WTnew in the acquisition training F(1,18)=0.9384, p=0.3455. (E) WT and WTnew showed a significant preference for the target quadrant in the probe trial (F) Swimming speed did not differ between WT and WTnew in the probe trial, p=0.1226. (G) Time to reach the target did not differ between WT and WTnew in the probe trial, p=0.9188. (H) Search strategies in the probe trial did not differ significantly between WT and WTnew control mice (chi-square, genotype: Day 1: p=0.3286, Day 2: p=0.2863, Day 3: p=0.8135, Day 4: p=0.5932, Day 5: p=0.8162)

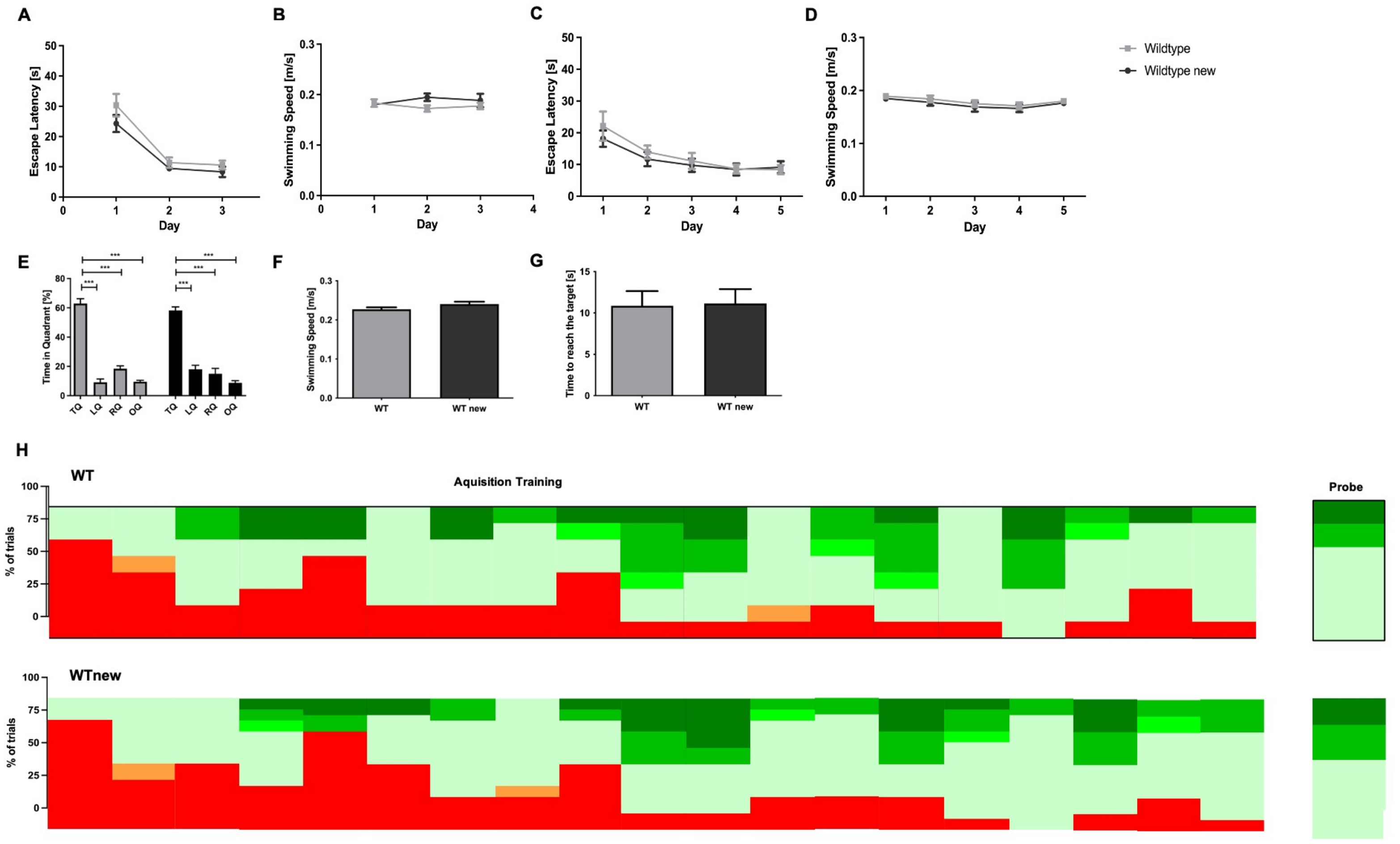

Supplement: Supplementary file 1 — Supplementary Information. [file 41598_2022_9270_MOESM1_ESM.pdf]
